# Abdominal bloating is the most bothersome symptom in irritable bowel syndrome with constipation (IBS-C): a large population-based Internet survey in Japan

**DOI:** 10.1186/s13030-016-0070-8

**Published:** 2016-06-04

**Authors:** Motoyori Kanazawa, Hiroto Miwa, Ayako Nakagawa, Masanori Kosako, Hiraku Akiho, Shin Fukudo

**Affiliations:** Department of Behavioral Medicine, Tohoku University Graduate School of Medicine, 2-1 Seiryo, Aoba, Sendai 980-8575 Japan; Division of Gastroenterology, Department of Internal Medicine, Hyogo College of Medicine, Nishinomiya, Japan; Japan-Asia Clinical Development 2, Astellas Pharma Inc, Tokyo, Japan

**Keywords:** Irritable bowel syndrome (IBS), Constipation, Abdominal bloating, Stress, Food, Epidemiology

## Abstract

**Background:**

Abdominal bloating is a common symptom in patients with irritable bowel syndrome with constipation (IBS-C). However, it is not included among the required items in the Rome III diagnostic criteria for IBS. Little is known about an impact of abdominal bloating seen in patients with IBS-C. Using a large population-based sample, the aim of the present study was to investigate what is the most bothersome symptom in subjects with IBS-C.

**Methods:**

An Internet survey of 30,000 adults drawn from the general public throughout Japan was conducted to identify subtypes of IBS using the Rome III diagnostic questionnaire. Consecutively, the screened subjects with IBS-C and the same number of age- and sex-matched non-IBS subjects who were randomly selected as controls were asked to answer a questionnaire on the degree of anxiety they experienced in their daily lives, thoughts about bowel habit, and their dominant gastrointestinal symptoms together with exacerbation factors (for IBS-C only).

**Results:**

The screening survey showed that the prevalence of overall IBS was 16.5 % (female 17.4 %, male 15.5 %) and that 2.8 % met the criteria for IBS-C, 4.5 % for IBS with diarrhea (IBS-D) and 8.2 % for mixed IBS (IBS-M). Seven hundred and fifty-nine of 835 (90.9 %) subjects with IBS-C and 746 of 830 (89.9 %) control subjects completed the consecutive questionnaire. IBS-C subjects felt a higher degree of anxiety in their daily lives (*p* < 0.01) and considered bowel habit to be an indicator of health (*p* < 0.01) to a greater extent than control subjects. In IBS-C, the degree of anxiety was significantly associated with abdominal discomfort (*p* < 0.01), pain (*p* < 0.01) and bloating (*p* = 0.02), but not with the frequency of bowel habit (*p* > 0.1). Abdominal bloating was the most bothersome symptom (27.5 %), which was more likely to occur after a meal (52.2 %), at work/school (29.2 %) and during times of stress (26.8 %). Only 4.5 % of IBS-C subjects reported abdominal pain as the ‘most bothersome’ symptom.

**Conclusions:**

A large population-based Internet survey suggests that abdominal bloating has a great impact on the daily lives of subjects diagnosed with IBS-C. Not only bowel movement/abdominal pain but also abdominal bloating should be evaluated in patients with IBS-C.

**Electronic supplementary material:**

The online version of this article (doi:10.1186/s13030-016-0070-8) contains supplementary material, which is available to authorized users.

## Background

Irritable bowel syndrome (IBS) is a common functional gastrointestinal (GI) disorder that affects between 10 and 20 % of the population worldwide [1]. Based on the Rome III diagnostic criteria for functional GI disorders, IBS is defined as recurrent abdominal pain or discomfort (at least 3 days per month in the last 3 months) associated with at least two of the following: improvement with defecation, onset associated with a change in stool frequency, and/or onset associated with change in stool form [2]. The diagnosis of IBS is subtyped by the predominant stool pattern: constipation (IBS-C), diarrhea (IBS-D), mixed (IBS-M), or unspecified (IBS-U) [2].

Approximately two-thirds of subjects with IBS relate their symptoms to their intake of food [3, 4]. Most of IBS patients modify their diet, and these modifications sometimes result in an inadequate diet [3] which may be associated with bowel symptoms. Patients with IBS also feel more stress compared to healthy subjects in their daily life [5, 6]. IBS symptoms often exaggerate when patients feel stress and/or anxiety [7]. Moreover, it has been reported that menstruation was associated with a worsening of abdominal pain and bloating and with more increase in rectal pain sensitivity compared with most other phases of the menstrual cycle in patients with IBS [8].

Abdominal bloating is commonly reported by patients with IBS [9]. However, it is not included among the required items in the Rome III diagnostic criteria [2]. Patients with IBS-C show more abdominal bloating and/or distention than those with IBS-D [10]. Constipation is often associated with delayed gastrointestinal transit [11]. The severity of abdominal bloating correlated strongly with the degree of abdominal distention only in IBS-C [12], suggesting that the pathophysiology is likely to be different between subtypes of bowel habit.

In general population, we have found that even non-consulters with IBS showed more impaired quality of life (QOL) compared to non-IBS subjects as well as patients with IBS [6, 13]. It has been reported that IBS symptom severity was associated with lower QOL [14, 15]. However, little is known about an impact of IBS symptoms including abdominal bloating seen in patients with IBS-C. Using a large population-based sample, the aim of the present study was to investigate what is the most bothersome symptom in subjects with IBS-C.

## Methods

As a preliminary survey, an Internet survey of 30,000 adults drawn from the general public throughout Japan (that is, from 47 prefectures in Japan) was conducted to identify subtypes of IBS. Participants were required to meet the following criteria to be eligible for the survey: (1) no conflicts of interest with advertising, marketing research, health care, or pharmaceutical/chemical companies; (2) 20-79 years of age; and (3) subjects who understand Japanese and were living in Japan. They were randomly selected using an online survey system (Automatic Internet Research System: AIRs, Macromill, Inc., Japan). Same numbers of males and females from the different five aged groups (20s, 30s, 40s, 50s and 60–79 years; 3000 subjects each) registered with the Macromill monitor panel (Macromill, Inc., Japan) were recruited during October 28–31, 2013 (Table 1).

**Table 1:** Demographics of study subjects (Preliminary study)

	Participants (*n* = 30,000), *n* (%)
Gender and age range (years)	
20-29 in females	3000 (10)
20-29 in males	3000 (10)
30-39 in females	3000 (10)
30-39 in males	3000 (10)
40-49 in females	3000 (10)
40-49 in males	3000 (10)
50-59 in females	3000 (10)
50-59 in males	3000 (10)
60-79 in females	3000 (10)
60-79 in males	3000 (10)
Marital status	
Married	17,391 (58)
Not married	12,609 (42)
Annual household income (yen)	
< 2 million	2557 (8.5)
2-4 million	6818 (22.7)
4-6 million	6564 (21.9)
6-8 million	3940 (13.1)
8-10 million	2321 (7.7)
10-12 million	1089 (3.6)
12-15 million	691 (2.3)
15-20 million	300 (1.0)
≧20 million	146 (0.5)
unknown	2795 (9.3)
not answered	2779 (9.3)
Diagnosis of IBS	
non-IBS	25,058 (83.5)
IBS (overall)	4942 (16.5)
IBS with constipation (IBS-C)	835 (2.8)
IBS with diarrhea (IBS-D)	1359 (4.5)
Mixed IBS (IBS-M)	2452 (8.2)
Unspecified IBS (IBS-U)	296 (1.0)

The IBS module on the Japanese version of the Rome III diagnostic questionnaire of which the validity and reliability have already been confirmed [16, 17] was used to identify IBS and subtypes of bowel habit (Additional file 1: Figure S1). IBS-C was defined when a patient reported hard or lumpy stools ≥25 % of the time, and loose or watery stools <25 % of the time, IBS-D was defined when a patient reported loose or watery stools ≥25 % of the time, and hard or lumpy stools <25 % of the time, IBS-M was defined when the patient reported loose or watery stools ≥25 % of the time, and hard or lumpy stools ≥25 % of the time, and IBS-U was defined when the patient could not be subtyped according to these criteria, but met all other diagnostic criteria for IBS [2]. Additionally, frequency of abdominal bloating/distention in the last 3 months was also asked. The online system guided participants to complete the questionnaire without remaining missing item answers.

Consequently, of the 30,000 participants, the screened subjects diagnosed as IBS-C and the same number of age- and sex-matched non-IBS subjects who were randomly selected as controls were invited to a main survey during November 1–4, 2013.

In the main survey, IBS-C subjects and controls were asked to answer a questionnaire which consists of 7 items on the degree of anxiety they experienced in their daily lives, number of bowel movement in a week and thoughts about bowel habit, and 6 items on their dominant GI symptoms (from the first to the third place) together with exacerbation factors such as situations and time periods in their daily lives (for IBS-C only, Additional file 2: Figure S2).

We conducted an anonymous Internet survey but regarded the online response as obtained informed consent for the present study. The disclosure of this study was approved by the Ethics Committee of Tohoku University School of Medicine (approval number: 2015-1-405).

The Mann–Whitney *U*-test or *χ*^2^ test was used for comparisons between two groups. The Kendall’s τ-b was used to evaluate associations between the symptoms and exacerbation factors among IBS-C. The level of statistical significance was set at a *P*-value of less than 0.05.

## Results

### The preliminary survey

The demographics of 30,000 participants were shown in Table 1. Mean age with standard deviation (SD) of the participants was 45 ± 14 years. The screening survey showed that the prevalence of overall IBS was 16.5 % (female 17.4 %, male 15.5 %) and that 2.8 % met the criteria for IBS-C, 4.5 % for IBS-D, 8.2 % for IBS-M, and 1.0 % for IBS-U. Subjects with IBS (*n* = 4942) were more likely to be female (53.0 vs. 49.4 %, *p* < 0.01) and were younger (42 ± 14 vs. 45 ± 15 years, *p* < 0.01) compared to non-IBS subjects (*n* = 25,058). IBS-C subjects alone (*n* = 835) were much more likely to be female (72.2 vs. 49.4 %, *p* < 0.01) but were older (47 ± 14 vs. 45 ± 15 years, *p* < 0.01) compared to non-IBS subjects. There was no significant difference in annual household income between IBS and non-IBS subjects. Subjects with IBS (overall, 70.0 %; IBS-C, 84.2 %; IBS-D, 53.3 %; IBS-M, 76.6 %; IBS-U, 51.7 %) were more likely to report abdominal bloating for at least two to three days a month compared to non-IBS subjects (19.5 %, *p* < 0.01, respectively).

### The main survey

Seven hundred and fifty-nine of 835 (90.9 %, 541 females) subjects with IBS-C and 746 of 830 (89.9 %, 537 females) age- and sex-matched subjects with non-IBS (controls) completed the consecutive questionnaire. IBS-C subjects felt a higher degree of anxiety in their daily lives (*p* < 0.01) and considered bowel habit to be an indicator of health (*p* < 0.01) to a greater extent than control subjects (Table 2).

**Table 2:** Comparisons of beliefs about bowel habit between IBS-C and controls (Main study)

	Controls (*n* = 746), *n* (%)	IBS-C (*n* = 759), *n* (%)	*p*-value
Female/male number	537/209 (72.0/28.0)	541/218 (71.3/28.7)	n.s.
Age (mean ± SD, years)	47 ± 14	47 ± 14	n.s.
Frequency of bowel habit (median, times/week)	7	3	<0.01
Ideal frequency of bowel habit			<0.01
6 times/week or less	113 (15.1)	165 (21.7)	
7 times/week	581 (77.9)	563 (74.2)	
8 times/week or more	52 (7.0)	31 (4.1)	
Considered bowel habit to be an indicator of health			<0.01
None	291 (39.0)	71 (9.4)	
Sometimes	206 (27.6)	221 (29.1)	
Often	90 (12.1)	186 (24.5)	
Mostly	69 (9.2)	148 (19.5)	
Always	90 (12.1)	133 (17.5)	
Degree of anxiety in daily life			<0.01
None	403 (54.0)	176 (23.2)	
Sometimes	283 (37.9)	440 (58.0)	
Often	46 (6.2)	111 (14.6)	
Almost	14 (1.9)	32 (4.2)	

The most common symptom associated with constipation in subjects with IBS-C was abdominal bloating (80.1 %) and was followed by excessive gas (71.3 %) and abdominal discomfort (64.3 %), whereas 29.1 % of IBS-C subjects reported abdominal pain during constipation (Table 3). The degree of anxiety was significantly associated with abdominal discomfort (Kendall’s τ = 0.09, *p* < 0.01), pain (τ = 0.11, *p* < 0.01), and bloating (τ = 0.08, *p* = 0.02), but not with the frequency of bowel habit (τ = -0.04, *p* > 0.1, Table 3).

**Table 3:** Associations between the degree of anxiety and GI symptoms in subjects with IBS-C

GI symptoms/Degree of anxiety	None	Sometimes	Often	Always	Kendall’s τ
Abdominal discomfort					0.09**
No (*n* = 271)	76	153	34	8	
Yes (*n* = 488, 64.3 %)	100	287	77	24	
Abdominal pain					0.11**
No (*n* = 538)	132	323	70	13	
Yes (*n* = 211, 29.1 %)	44	117	41	19	
Abdominal bloating					0.08*
No (*n* = 151)	42	91	13	5	
Yes (*n* = 608, 80.1 %)	134	349	98	27	
Abdominal distention					0.11**
No (*n* = 368)	94	224	39	11	
Yes (*n* = 391, 51.5 %)	82	216	72	21	
Sensation of excessive gas					0.11**
No (*n* = 218)	65	122	25	6	
Yes (*n* = 541, 71.3 %)	111	318	86	26	
Tightness in the abdomen					0.11**
No (*n* = 408)	108	237	52	11	
Yes (*n* = 351, 46.2 %)	68	203	59	21	
Abdominal fullness					0.08*
No (*n* = 636)	157	364	92	23	
Yes (*n* = 123, 16.2 %)	19	76	19	9	
Borborygmus					0.06
No (*n* = 626)	148	367	91	20	
Yes (*n* = 133, 17.5 %)	28	73	20	12	
Increase in passing of gas					0.08*
No (*n* = 369)	99	208	48	14	
Yes (*n* = 390, 51.4 %)	77	232	63	18	
Decrease in passing of gas					0.01
No (*n* = 736)	172	425	109	30	
Yes (*n* = 23, 3.0 %)	4	15	2	2	
Incomplete evacuation					0.00
No (*n* = 326)	72	198	41	15	
Yes (*n* = 433, 57.0 %)	104	242	70	17	
Urgency					0.04
No (*n* = 673)	156	397	95	25	
Yes (*n* = 86, 11.3 %)	20	43	16	7	
Sensation of anorectal obstruction/blockage					0.01
No (*n* = 348)	86	194	54	14	
Yes (*n* = 411, 54.2 %)	90	246	111	18	
Frequency of bowel habit in a week					−0.04
> 1 time (*n* = 15)	3	8	3	1	
1 time (*n* = 60)	6	39	12	3	
2 times (*n* = 156)	37	94	19	6	
3 times (*n* = 152)	40	78	25	9	
4 times (*n* = 85)	22	49	11	3	
5 times (*n* = 79)	17	48	11	3	
6 times (*n* = 52)	12	31	7	2	
7 times (*n* = 111)	26	61	20	4	
8 times or more (*n* = 49)	13	32	3	1	

*p < 0.05, **p < 0.01, the Kendall’s τ-b

**Fig. 1:**
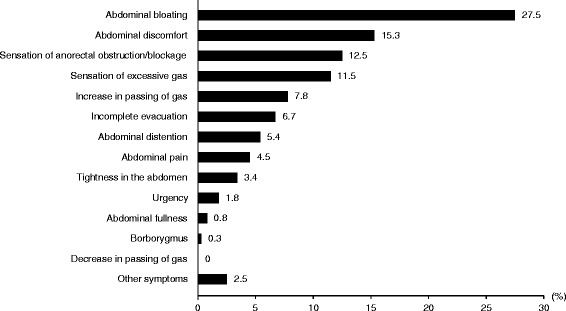
The frequencies of the most bothersome symptom reported by subjects with IBS-C (*n* = 759)

As shown in Fig. 1 and Table 4, the most bothersome symptom in subjects with IBS-C was abdominal bloating (27.5 %, 163 females and 46 males), which was more likely to occur after a meal (52.2 %), at work/school (29.2 %), during times of stress (26.8 %) and during menstruation (11.7 %, females only). Only 4.5 % of IBS-C subjects (25 females and 9 males) reported abdominal pain as the most bothersome symptom, which was more likely to occur after a meal (26.5 %), at work/school (29.4 %), during times of stress (29.4 %) and during menstruation (48.0 %, females only). IBS-C subjects were more likely to report that the bothersome symptoms usually occur throughout the day or during a non-specific period of time rather than at a regular time during the day (data were not shown).

**Table 4:** Occurrence situations of the ‘most bothersome’ symptom in subjects with IBS-C

Situations/Symptoms	Abdominal bloating (*n* = 209)	Abdominal discomfort (*n* = 116)	Sensation of anorectal obstruction/blockage (*n* = 95)	Sensation of excessive gas (*n* = 87)	Increase in passing of gas (*n* = 59)	Incomplete evacuation (*n* = 51)	Abdominal distention (*n* = 41)	Abdominal pain (*n* = 34)
On the way to work/school by bus or train	18.2	21.6	4.2	23.0	35.6	9.8	14.6	5.9
At work/school	29.2	25.9	11.6	43.7	45.8	17.6	31.7	29.4
During a conference presentation/an exam	5.7	10.3	2.1	9.2	5.1	3.9	7.3	8.8
After drinking alcohol	2.4	6.9	3.2	5.7	6.8	0	2.4	0
After drinking milk	2.9	5.2	3.2	4.6	3.4	0	4.9	11.8
After a meal	52.2	43.1	22.1	26.4	39.0	21.6	43.9	26.5
On a sightseeing trip	24.4	13.8	20.0	18.4	11.9	11.8	14.6	8.8
On a business trip	3.3	6.9	6.3	3.4	1.7	2.0	7.3	2.9
During time of stress	26.8	31.9	25.3	28.7	25.4	27.5	31.7	29.4
After taking some drugs	2.9	1.7	8.4	0	0	0	2.4	5.9
During menstruation (females only)	11.7	16.2	8.3	4.6	12.2	11.4	3.6	48.0

Only symptoms which at least 30 subjects reported were shown. Data were expressed as frequencies (%) in each symptom

## Discussion

We conducted a large-scale Internet survey investigating 30,000 adults from the general public throughout Japan. In this study, we estimated frequencies of the subtypes of IBS using the Rome III diagnostic questionnaire [16, 17], and then investigated what is the most bothersome symptom and what factors in the daily life exaggerate the dominant symptoms among subjects with IBS-C. As long as we know, this is the first report on a large number of subjects diagnosed as IBS-C demonstrating that the degree of anxiety in their daily lives is not always associated with frequency of bowel movement. Furthermore, we also found that the most bothersome symptom, abdominal bloating, occurs most often after meal, subsequently at work or school and during time of stress in subjects with IBS-C.

The sensation of abdominal bloating may affect 10 to 30 % of healthy subjects and up to 96 % of patients with IBS [9]. Abdominal bloating is more often noted by females with IBS than males, with symptoms increasing in relation to menses [18]. Abdominal bloating is associated with decreased QOL and may cause a higher number of physician visits [19]. Indeed, the IBS symptom severity scale (IBS-SSS) [20], which is widely used to assess the severity of IBS, consists of the item on the severity score for abdominal bloating as well as abdominal pain. However, the presence of abdominal bloating in other functional GI disorders and even in healthy subjects [19] means that it is not necessarily considered a diagnostic criterion for IBS.

‘Abdominal bloating’ is not always a specific term that implies both to the subjective sensation and to the objective or ‘visible’ abdominal distention [21]. In the present study, the symptom descriptions in Japanese, ‘abdominal bloating’, ‘abdominal distention’, ‘sensation of excessive gas’, ‘tightness in the abdomen’ and ‘abdominal fullness’ were used as independent symptomatic items on the main questionnaire. In our findings, the most common symptom of these 5 terms which IBS subjects expressed during constipation was abdominal bloating and the fewest was abdominal fullness (see Table 3).

Previous findings of abdominal girth using plethysmography [21, 22] may support our results of different frequencies of abdominal bloating and distention in subjects with IBS-C. Using this technique it has been demonstrated that most patients who have abdominal distention also report abdominal bloating and that patients with bloating alone have lower sensory thresholds in the rectum compared to healthy subjects, whereas those with abdominal bloating and distention have normal or slightly higher sensory thresholds [21]. On the other hand, it has been reported that increase in abdominal girth in a day directly correlated with orocecal and colonic transit times, and inversely with stool consistency [22]. Therefore, abdominal bloating alone may be more reflection of visceral hypersensitivity, whereas abdominal bloating with distension may be more of a peripheral physiological problem.

In our subjects with IBS-C, the degree of anxiety in their daily lives was associated with severity of abdominal pain, discomfort and bloating, but not with frequency of bowel movement. It has been demonstrated that IBS patients feel more stress which may exaggerate IBS symptoms such as abdominal pain compared to healthy subjects [5]. In patients with IBS, psychological or physiological stimulus (i.e., stress) alters both visceral perception (mostly, hypersensitivity) [23–25] and colonic motility (mostly, hypercontractility even in IBS-C) [26, 27]. However, it has been reported that only 11.5 % of patients with IBS-C showed delayed colonic transit [11]. In addition, no relationship has been detected between stress factors and colonic transit in IBS patients [28]. These findings suggest that lower frequency of bowel movement in IBS-C may not always reflect inhibition of bowel movement by stress.

IBS patients often relate their symptoms to the intake of certain foods. IBS symptoms such as abdominal pain and bloating occur or are exacerbated postprandially in approximately two-thirds of patients [3, 4]. We have previously reported that female subjects with IBS had more irregular meals and skipped meals more frequently compared to non-IBS subjects in nursing and medical school students [29]. Furthermore, IBS patients showed an exaggerated sensory component of the gastrocolonic response, expressed as lowered colonic perception thresholds and increased viscerosomatic referral area for abdominal pain after duodenal lipid infusion [30]. On the other hand, an intestinal transit study using scintigraphy revealed that IBS-C patients showed normal ileocolonic transit but somewhat delayed colonic transit in response to meal ingestion [31]. We have also demonstrated that meal ingestion did not induce altered colonic motility response in patients with IBS-C [27]. Therefore, exaggerated abdominal boating in IBS-C subjects after the meals in this study may be due to increased visceral perception, increased gut contractions and impaired propulsive movement in response to the nutritions.

A limitation of our study was to use the Internet-based survey. Subjects who were interested in own health might be more likely to participate in this survey. In the present study, 30,000 samples were collected from the large monitor panel throughout Japan and set same numbers of sex and several aged group flames. Therefore, we believe ascertainment bias is unlikely because the prevalence of overall IBS and IBS-C using the Rome III diagnostic criteria was similar to what has been reported previously for Japan [32, 33] or the other countries [34, 35]. Another limitation was that we did not investigate whether the presence of an organic GI disease and/or the other comorbid disease. It has been reported that IBS patients have more somatic/psychiatric comorbidities which may affect their daily lives compared to non-IBS subjects [36].

To summarize, a large population-based Internet survey has shown that a number of similarities between the Western countries and Japan, including a similar prevalence of IBS-C and a similar frequency of abdominal bloating in subjects with IBS-C. We have also shown important similarities with the previous surveys including a similar association of severity of abdominal pain with the degree of anxiety and a similar association of occurrence/exaggeration of IBS symptoms with meal ingestion and with perceived stress. Thus, these trigger factors are considered to be generalizable even in IBS-C. In constant, a significant association of frequency of bowel movement with the degree of anxiety has failed to be observed in IBS-C subjects.

## Conclusions

Our findings suggest that abdominal bloating has a great impact on the daily lives of subjects diagnosed with IBS-C. Not only bowel movement/abdominal pain but also abdominal bloating should be evaluated in patients with IBS-C.

## Abbreviations

GI, gastrointestinal; IBS, irritable bowel syndrome; IBS-C, IBS with constipation; IBS-D, IBS with diarrhea; IBS-M, mixed IBS; IBS-U, unspecified IBS; QOL, quality of life.

## Additional files

Additional file 1: Figure S1. Online questionnaire for the preliminary study (written in Japanese). (PDF 380 kb) Additional file 2: Figure S2. Online questionnaire for the main study (written in Japanese). (PDF 1000 kb)
