# Supplementary material for: Abdominal bloating is the most bothersome symptom in irritable bowel syndrome with constipation (IBS-C): a large population-based Internet survey in Japan
Source: Biopsychosoc Med. 2016 Jun 4;10:19. doi: 10.1186/s13030-016-0070-8 (PMC4893246; doi:10.1186/s13030-016-0070-8)
Supplement: Additional file 1: Figure S1. — Online questionnaire for the preliminary study (written in Japanese). (PDF 380 kb) [file 13030_2016_70_MOESM1_ESM.pdf]

MACROMILL

あなたご自身に関するアンケート

当アンケートでは「あなたの健康状態」について
お伺いする箇所が含まれております。
本件趣旨にご同意くださる方は、ご回答をお願いいたします。

回答をしたくないと判断された場合はお手数ですが、
「回答をやめる」ボタン、あるいはブラウザを閉じて、アンケートを終了してください。

なお、当アンケートにより取得した回答結果につきましては、
市場の実態把握のために特定の個人が識別できないよう全て統計的に処理し、
活用させていただきます。

下記アンケートにご協力お願いいたします。

当アンケートの回答者の皆様へお願い

マクロミルモニタの皆様にはモニタ規約にて「調査についての守秘義務」の徹底をお願いしています。

当アンケートの内容および当アンケートで知り得た情報については、決して第三者に口外しないよう（掲示板やホームページへの書き込みを含む）、ご協力お願いします。

今現在、お薬（下剤など）をのまれている方は、のみ始める前の状態（調子が悪かったときの状態）を 思い起こしてお答え下さい。

Q1 最近3ヶ月間に、おなかのどこかの不快感、または、痛みが、どのくらいありましたか？
【 必須入力 】

- ○ 0. なし
- ○ 1. 月に1日未満
- ○ 2. 月に1日
- ○ 3. 月に2日か3日
- ○ 4. 週に1日
- ○ 5. 週に1日より多い
- ○ 6. 毎日

ここで改ページ

今現在、お薬（下剤など）をのまれている方は、のみ始める前の状態（調子が悪かったときの状態）を 思い起こしてお答え下さい。

Q2 女性へ：この不快感や痛みは、月経の間だけあって、他の時には起きませんか？
男性へ：【2】の答えを選んで次に進んでください。
【 必須入力 】

- ○ 0. いいえ
- ○ 1. はい
- ○ 2. あてはまらない、なぜなら生理が変わった（閉経） あるいは、男性であるから

ここで改ページ

今現在、お薬（下剤など）をのまれている方は、のみ始める前の状態（調子が悪かったときの状態）を 思い起こしてお答え下さい。

Q3 この不快感や痛みは、6ヶ月以上前からありましたか？
【 必須入力 】

- ○ 0. いいえ
- ○ 1. はい

ここで改ページ

今現在、お薬（下剤など）をのまれている方は、のみ始める前の状態（調子が悪かったときの状態）を 思い起こしてお答え下さい。

Q4 以下の項目であてはまるものをそれぞれお選びください。
【 必須入力 】

		0 なし、あるいはまれ	1 ときどき	2 しばしば	3 ほとんどの時	4 いつも
0. この不快感や痛みは、便が出ると良くなるか、なくなるということが、どのくらいありましたか？	→	○	○	○	○	○
1. この不快感や痛みがはじまる時は、便を出す回数が多くなりましたか？	→	○	○	○	○	○
2. この不快感や痛みがはじまる時は、便を出す回数が少なくなりましたか？	→	○	○	○	○	○
3. この不快感や痛みがはじまる時は、便がよりゆるくなりましたか？	→	○	○	○	○	○
4. この不快感や痛みがはじまる時は、便がより硬くなりましたか？	→	○	○	○	○	○

ここで改ページ

今現在、お薬（下剤など）をのまれている方は、のみ始める前の状態（調子が悪かったときの状態）を 思い起こしてお答え下さい。

Q5 最近3ヶ月間に、おなかが張る感じ、または、おなかが膨らむことが、どのくらいありましたか？
【 必須入力 】

- ○ 0. なし
- ○ 1. 月に1日未満
- ○ 2. 月に1日
- ○ 3. 月に2日か3日
- ○ 4. 週に1日
- ○ 5. 週に1日より多い
- ○ 6. 毎日

ここで改ページ

今現在、お薬（下剤など）をのまれている方は、のみ始める前の状態（調子が悪かったときの状態）を 思い起こしてお答え下さい。

Q6 最近3ヶ月間に、かたい便や、コロコロした便が出ることが、どのくらいありましたか？
【 必須入力 】

- ○ 0. なし、あるいはまれ
- ○ 1. 全体の約25%
- ○ 2. 全体の約50%
- ○ 3. 全体の約75%
- ○ 4. いつも、全体の約100%

ここで改ページ

今現在、お薬（下剤など）をのまれている方は、のみ始める前の状態（調子が悪かったときの状態）を 思い起こしてお答え下さい。

Q7 最近3ヶ月間に、くずれた便、泥のような便、あるいは水のような便が出ることが、どのくらいありましたか？
【 必須入力 】

- ○ 0. なし、あるいはまれ
- ○ 1. 全体の約25%
- ○ 2. 全体の約50%
- ○ 3. 全体の約75%
- ○ 4. いつも、全体の約100%

アンケートは以上で終わりです。ご協力ありがとうございました。
回答もれがないか確認し、よろしければ「送信」ボタンをクリックしてください。

送　信

© MACROMILL, INC.
