# Supplementary material for: Abdominal bloating is the most bothersome symptom in irritable bowel syndrome with constipation (IBS-C): a large population-based Internet survey in Japan
Source: Biopsychosoc Med. 2016 Jun 4;10:19. doi: 10.1186/s13030-016-0070-8 (PMC4893246; doi:10.1186/s13030-016-0070-8)
Supplement: Additional file 2: Figure S2. — Online questionnaire for the main study (written in Japanese). (PDF 1000 kb) [file 13030_2016_70_MOESM2_ESM.pdf]

MACROMILL

健康に関するアンケート

当アンケートでは「あなたの健康状態」について
お伺いする箇所が含まれております。
本件趣旨にご同意くださる方は、ご回答をお願いいたします。

回答をしたくないと判断された場合はお手数ですが、
「回答をやめる」ボタン、あるいはブラウザを閉じて、アンケートを終了してください。

なお、当アンケートにより取得した回答結果につきましては、
市場の実態把握のために特定の個人が識別できないよう全て統計的に処理し、
活用させていただきます。

下記アンケートにご協力お願いいたします。

当アンケートの回答者の皆様へお願い

マクロミルモニタの皆様にはモニタ規約にて「調査についての守秘義務」の徹底をお願いしています。

当アンケートの内容および当アンケートで知り得た情報については、決して第三者に口外しないよう(掲示板やホームページへの書き込みを含む)、ご協力お願いします。

AC 割付条件設定（回答者には表示されません）

- ☐ 1. IBS-C
- ☐ 2. Normal

Q1 ここ1週間のあいだに、緊張している、あるいは「ぴりぴりしている」と感じたことがありますか？

【 必須入力 】

- ○ 0. ほとんどいつも
- ○ 1. 多くの時間
- ○ 2. ときどき
- ○ 3. まったくない

ここで改ページ

ここからの質問は、あなたのおなかの症状についてお聞きするものです。
それぞれの質問について最もよく当てはまるところをお知らせください。
今現在、お薬（下剤など）をのまれている方は、
のみ始める前の状態（調子が悪かったときの状態）を思い起こしてお答え下さい。

Q2 以下の項目であてはまるものをそれぞれお選びください。

【 必須入力 】

	0 なし、あるいはまれ	1 ときどき	2 しばしば	3 ほとんどの時	4 いつも
0. 最近3ヶ月間に、便を出す回数が1週間に3回未満（0-2回）であることが、どのくらいありましたか？ →	○	○	○	○	○
1. 最近3ヶ月間に、便を出すときにりきむことが、どのくらいありましたか？ →	○	○	○	○	○
2. 最近3ヶ月間に、便を出しきるために、下腹部やそのまわりを押したり便を取り除いたことが、どのくらいありましたか？ →	○	○	○	○	○
3. 最近3ヶ月間に、便を出す時に、肛門をゆるめたり便を外に出しにくかったことが、どのくらいありましたか？ →	○	○	○	○	○
4. 最近3ヶ月間に、排便時に、粘液やねばねばする液が、どのくらいありましたか？ →	○	○	○	○	○

ここで改ページ

今現在、お薬（下剤など）をのまれている方は、
のみ始める前の状態（調子が悪かったときの状態）を思い起こしてお答え下さい。

Q3 便の調子が健康状態に反映されていると思いますか。

【 必須入力 】

- ○0. なし、あるいはまれに思う
- ○1. ときどき思う
- ○2. しばしば思う
- ○3. ほとんどの時に思う
- ○4. いつも思う

ここで改ページ

今現在、お薬（下剤など）をのまれている方は、
のみ始める前の状態（調子が悪かったときの状態）を思い起こしてお答え下さい。

Q4 普段は1週間で何回くらい便を出しますか。

【 必須入力 】

- ○0. 1回未満（1週間出ない）→具体的に：[　　　]日に1回（半角数字）
- ○1. 1回
- ○2. 2回
- ○3. 3回
- ○4. 4回
- ○5. 5回
- ○6. 6回
- ○7. 7回
- ○8. 8回以上→具体的に：[　　　]回（半角数字）

ここで改ページ

今現在、お薬（下剤など）をのまれている方は、
のみ始める前の状態（調子が悪かったときの状態）を思い起こしてお答え下さい。

Q5 何日くらいの間に1回便が出ないと便秘だと思いますか。

【 必須入力 】

- ○0. 1日複数回
- ○1. 1日
- ○2. 2日
- ○3. 3日
- ○4. 4日
- ○5. 5日
- ○6. 6日
- ○7. 7日
- ○8. 8日以上→具体的に：[　　　]日（半角数字）

ここで改ページ

今現在、お薬（下剤など）をのまれている方は、
のみ始める前の状態（調子が悪かったときの状態）を思い起こしてお答え下さい。

Q6 何日くらいに1回便が出ないと不安に感じますか。

【 必須入力 】

- ○0. 1日複数回
- ○1. 1日
- ○2. 2日
- ○3. 3日
- ○4. 4日
- ○5. 5日
- ○6. 6日
- ○7. 7日
- ○8. 8日以上→具体的に：[　　　]日（半角数字）

ここで改ページ

今現在、お薬（下剤など）をのまれている方は、
のみ始める前の状態（調子が悪かったときの状態）を思い起こしてお答え下さい。

Q7 1週間で何回くらい便を出すことが理想ですか。

【 必須入力 】

- ○0. 1回未満
- ○1. 1回
- ○2. 2回
- ○3. 3回
- ○4. 4回
- ○5. 5回
- ○6. 6回
- ○7. 7回
- ○8. 8回以上→具体的に：[　　　]回（半角数字）

●●●●●●●●●●●●●●●●●●●● ✂ ここで改ページ ●●●●●●●●●●●●●●●●●●●●

今現在、お薬（下剤など）をのまれている方は、
のみ始める前の状態（調子が悪かったときの状態）を思い起こしてお答え下さい。

Q8 便秘のときに起こるあなたの困っている症状についてお聞きします。
あてはまる症状を、すべてお知らせください。（いくつでも）
※その他をお答えになる場合は、自由回答欄1つにつき1つの症状をお答えください。

【 必須入力 】

- □0. おなかの不快感がある
- □1. おなかの痛みがある
- □2. おなかが張る感じがある
- □3. おなかが膨らむことがある
- □4. おなかにガスがたまっている感じがある
- □5. おなかがきつい感じがある
- □6. いつも満腹な感じがある
- □7. おなかがごろごろと鳴る
- □8. おならの回数が増える
- □9. おならの回数が減る
- □10. 便を出した後も便が残っている感じがある
- □11. 便意のため急いでトイレにかけこまなければならないことがある
- □12. 便を出しているときに、便を出しにくい感じ（ふさがる感じ）がある
- □13. その他1[　　　　　　]
- □14. その他2[　　　　　　]
- □15. その他3[　　　　　　]

●●●●●●●●●●●●●●●●●●●● ✂ ここで改ページ ●●●●●●●●●●●●●●●●●●●●

今現在、お薬（下剤など）をのまれている方は、
のみ始める前の状態（調子が悪かったときの状態）を思い起こしてお答え下さい。

Q9 Q8でお答えいただいた便秘のときに起こる症状の中で、あなたが困っていて、
よく表している症状を3つまで順位をつけてください。
※この設問は縦方向にお答えください。
※前問の回答により2位までしか表示されない場合があります。 その際は、2位までお答えください。

【 必須入力 】

	1位<br>↓	2位<br>↓	3位<br>↓
0. おなかの不快感がある	○	○	○
1. おなかの痛みがある	○	○	○
2. おなかが張る感じがある	○	○	○
3. おなかが膨らむことがある	○	○	○
4. おなかにガスがたまっている感じがある	○	○	○
5. おなかがきつい感じがある	○	○	○
6. いつも満腹な感じがある	○	○	○
7. おなかがごろごろと鳴る	○	○	○
8. おならの回数が増える	○	○	○
9. おならの回数が減る	○	○	○
10. 便を出した後も便が残っている感じがある	○	○	○
11. 便意のため急いでトイレにかけこまなければならないことがある	○	○	○
12. 便を出しているときに、便を出しにくい感じ（ふさがる感じ）がある	○	○	○
13. その他1【【Q8_14FAの選択内容】】	○	○	○
14. その他2【【Q8_15FAの選択内容】】	○	○	○
15. その他3【【Q8_16FAの選択内容】】	○	○	○

ここで改ページ

今現在、お薬（下剤など）をのまれている方は、
のみ始める前の状態（調子が悪かったときの状態）を思い起こしてお答え下さい。

Q10 Q9でお答えいただいた便秘に伴う症状の程度について、いずれか一つをお知らせください。

【 必須入力 】

		0 非常に弱い	1 弱い	2 中程度	3 強い	4 非常に強い
0. おなかの不快感がある	→	○	○	○	○	○
1. おなかの痛みがある	→	○	○	○	○	○
2. おなかが張る感じがある	→	○	○	○	○	○
3. おなかが膨らむことがある	→	○	○	○	○	○
4. おなかにガスがたまっている感じがある	→	○	○	○	○	○
5. おなかがきつい感じがある	→	○	○	○	○	○
6. いつも満腹な感じがある	→	○	○	○	○	○
7. おなかがごろごろと鳴る	→	○	○	○	○	○
8. おならの回数が増える	→	○	○	○	○	○
9. おならの回数が減る	→	○	○	○	○	○
10. 便を出した後も便が残っている感じがある	→	○	○	○	○	○
11. 便意のため急いでトイレにかけこまなければならないことがある	→	○	○	○	○	○
12. 便を出しているときに、便を出しにくい感じ（ふさがる感じ）がある	→	○	○	○	○	○
13. その他1【【Q8_14FAの選択内容】】	→	○	○	○	○	○
14. その他2【【Q8_15FAの選択内容】】	→	○	○	○	○	○
15. その他3【【Q8_16FAの選択内容】】	→	○	○	○	○	○

ここで改ページ

今現在、お薬（下剤など）をのまれている方は、
のみ始める前の状態（調子が悪かったときの状態）を思い起こしてお答え下さい。

Q11 Q9でお答えいただいた便秘に伴う症状はどんな状況で起こりますか。
あてはまる状況を、すべてお知らせください。（それぞれいくつでも）

【 必須入力 】

		0 電車やバスなど通勤・通学途中	1 就業・就労・授業中	2 会議での発表や試験を受けるとき	3 お酒を飲んだとき	4 牛乳を飲んだとき	5 食事をとったとき	6 旅行に出かけたとき	7 出張に出かけたとき	8 何らかのストレスを感じたとき	9 他の病気の薬を飲んだとき	10 月経の間	11 その他
0. おなかの不快感がある	→	□	□	□	□	□	□	□	□	□	□	□	
1. おなかの痛みがある	→	□	□	□	□	□	□	□	□	□	□	□	
2. おなかが張る感じがある	→	□	□	□	□	□	□	□	□	□	□	□	
3. おなかが膨らむことがある	→	□	□	□	□	□	□	□	□	□	□	□	
4. おなかにガスがたまっている感じがある	→	□	□	□	□	□	□	□	□	□	□	□	
5. おなかがきつい感じがある	→	□	□	□	□	□	□	□	□	□	□	□	
6. いつも満腹な感じがある	→	□	□	□	□	□	□	□	□	□	□	□	
7. おなかがごろごろと鳴る	→	□	□	□	□	□	□	□	□	□	□	□	
8. おならの回数が増える	→	□	□	□	□	□	□	□	□	□	□	□	
9. おならの回数が減る	→	□	□	□	□	□	□	□	□	□	□	□	
10. 便を出した後も便が残っている感じがある	→	□	□	□	□	□	□	□	□	□	□	□	
11. 便意のため急いでトイレにかけこまなければならないことがある	→	□	□	□	□	□	□	□	□	□	□	□	
12. 便を出しているときに、便を出しにくい感じ（ふさがる感じ）がある	→	□	□	□	□	□	□	□	□	□	□	□	
13. その他1【【Q8_14FAの選択内容】】	→	□	□	□	□	□	□	□	□	□	□	□	
14. その他2【【Q8_15FAの選択内容】】	→	□	□	□	□	□	□	□	□	□	□	□	
15. その他3【【Q8_16FAの選択内容】】	→	□	□	□	□	□	□	□	□	□	□	□	

ここで改ページ

今現在、お薬（下剤など）をのまれている方は、
のみ始める前の状態（調子が悪かったときの状態）を思い起こしてお答え下さい。

Q12 **Q11でお答えいただいた状況の中で、一番困っている状況を一つお知らせください。（それぞれひとつずつ）**

【 必須入力 】

		0 電車やバスなど通勤・通学途中	1 就業・就労・授業中	2 会議での発表や試験を受けるとき	3 お酒を飲んだとき	4 牛乳を飲んだとき	5 食事をとったとき	6 旅行に出かけたとき	7 出張に出かけたとき	8 何らかのストレスを感じたとき	9 他の病気の薬を飲んだとき	10 月経の間	11 その他
0. おなかの不快感がある	→	○	○	○	○	○	○	○	○	○	○	○	○
1. おなかの痛みがある	→	○	○	○	○	○	○	○	○	○	○	○	○
2. おなかが張る感じがある	→	○	○	○	○	○	○	○	○	○	○	○	○
3. おなかが膨らむことがある	→	○	○	○	○	○	○	○	○	○	○	○	○
4. おなかにガスがたまっている感じがある	→	○	○	○	○	○	○	○	○	○	○	○	○
5. おなかがきつい感じがある	→	○	○	○	○	○	○	○	○	○	○	○	○
6. いつも満腹な感じがある	→	○	○	○	○	○	○	○	○	○	○	○	○
7. おなかがごろごろと鳴る	→	○	○	○	○	○	○	○	○	○	○	○	○
8. おならの回数が増える	→	○	○	○	○	○	○	○	○	○	○	○	○
9. おならの回数が減る	→	○	○	○	○	○	○	○	○	○	○	○	○
10. 便を出した後も便が残っている感じがある	→	○	○	○	○	○	○	○	○	○	○	○	○
11. 便意のため急いでトイレにかけこまなければならないことがある	→	○	○	○	○	○	○	○	○	○	○	○	○
12. 便を出しているときに、便を出しにくい感じ（ふさがる感じ）がある	→	○	○	○	○	○	○	○	○	○	○	○	○
13. その他1【【Q8_14FAの選択内容】】	→	○	○	○	○	○	○	○	○	○	○	○	○
14. その他2【【Q8_15FAの選択内容】】	→	○	○	○	○	○	○	○	○	○	○	○	○
15. その他3【【Q8_16FAの選択内容】】	→	○	○	○	○	○	○	○	○	○	○	○	○

ここで改ページ

今現在、お薬（下剤など）をのまれている方は、
のみ始める前の状態（調子が悪かったときの状態）を思い起こしてお答え下さい。

Q13 **Q9でお答えいただいた便秘に伴う症状があらわれるのは1日のうち、どの時間帯が一番多いですか。**

【 必須入力 】

		0 午前	1 午後	2 夕方	3 夜間	4 1日を通してずっと	5 決まった時間帯はない
0. おなかの不快感がある	→	○	○	○	○	○	○
1. おなかの痛みがある	→	○	○	○	○	○	○
2. おなかが張る感じがある	→	○	○	○	○	○	○
3. おなかが膨らむことがある	→	○	○	○	○	○	○
4. おなかにガスがたまっている感じがある	→	○	○	○	○	○	○
5. おなかがきつい感じがある	→	○	○	○	○	○	○
6. いつも満腹な感じがある	→	○	○	○	○	○	○
7. おなかがごろごろと鳴る	→	○	○	○	○	○	○
8. おならの回数が増える	→	○	○	○	○	○	○
9. おならの回数が減る	→	○	○	○	○	○	○
10. 便を出した後も便が残っている感じがある	→	○	○	○	○	○	○
11. 便意のため急いでトイレにかけこまなければならないことがある	→	○	○	○	○	○	○
12. 便を出しているときに、便を出しにくい感じ（ふさがる感じ）がある	→	○	○	○	○	○	○
13. その他1【【Q8_14FAの選択内容】】	→	○	○	○	○	○	○
14. その他2【【Q8_15FAの選択内容】】	→	○	○	○	○	○	○
15. その他3【【Q8_16FAの選択内容】】	→	○	○	○	○	○	○

アンケートは以上で終わりです。ご協力ありがとうございました。
回答もれがないか確認し、よろしければ「送信」ボタンをクリックしてください。

送　信

© MACROMILL, INC.
